# Supplementary material for: Large household reduces dementia mortality: A cross-sectional data analysis of 183 populations
Source: PLoS One. 2022 Mar 3;17(3):e0263309. doi: 10.1371/journal.pone.0263309 (PMC8893634; doi:10.1371/journal.pone.0263309)
Supplement: Attachment — Submitted filename: 1. PLOS ONE 1 to 1 Response.docx [file pone.0263309.s002.docx]

PLOS ONE Decision: Revision required [PONE-D-21-26012] - [EMID: a9dd082ffa229339]

PONE-D-21-26012

Large household reduces dementia mortality: indications for patient care

PLOS ONE

Dear Dr. Wenpeng You,

Thank you for submitting your manuscript to PLOS ONE. After careful consideration, we feel that it has merit but does not fully meet PLOS ONE’s publication criteria as it currently stands. Therefore, we invite you to submit a revised version of the manuscript that addresses the points raised during the review process.

Please submit your revised manuscript by OCT 10, 2021. If you will need more time than this to complete your revisions, please reply to this message or contact the journal office at plosone@plos.org. When you're ready to submit your revision, log on to <https://www.editorialmanager.com/pone/> and select the 'Submissions Needing Revision' folder to locate your manuscript file.

Please include the following items when submitting your revised manuscript:

• A rebuttal letter that responds to each point raised by the academic editor and reviewer(s). You should upload this letter as a separate file labeled 'Response to Reviewers'.

• A marked-up copy of your manuscript that highlights changes made to the original version. You should upload this as a separate file labeled 'Revised Manuscript with Track Changes'.

• An unmarked version of your revised paper without tracked changes. You should upload this as a separate file labeled 'Manuscript'.

If you would like to make changes to your financial disclosure, please include your updated statement in your cover letter. Guidelines for resubmitting your figure files are available below the reviewer comments at the end of this letter.

If applicable, we recommend that you deposit your laboratory protocols in protocols.io to enhance the reproducibility of your results. Protocols.io assigns your protocol its own identifier (DOI) so that it can be cited independently in the future. For instructions see: <https://journals.plos.org/plosone/s/submission-guidelines#loc-laboratory-protocols>. Additionally,

PLOS ONE offers an option for publishing peer-reviewed Lab Protocol articles, which describe protocols hosted on protocols.io. Read more information on sharing protocols at <https://plos.org/protocols?utm_medium=editorial-email&utm_source=authorletters&utm_campaign=protocols>.

We look forward to receiving your revised manuscript.

Kind regards,

Wen-Wei Sung, M.D., Ph.D.

Academic Editor

PLOS ONE

Journal requirements:

When submitting your revision, we need you to address these additional requirements.

1. Please ensure that your manuscript meets PLOS ONE's style requirements, including those for file naming. The PLOS ONE style templates can be found at

https://journals.plos.org/plosone/s/file?id=wjVg/PLOSOne_formatting_sample_main_body.pdf and

<https://journals.plos.org/plosone/s/file?id=ba62/PLOSOne_formatting_sample_title_authors_affiliations.pdf>

2. We noticed you have some minor occurrence of overlapping text with the following previous publication(s), which needs to be addressed:

- <https://file.scirp.org/Html/1-2470163_81611.htm>

The text that needs to be addressed involves the Results section.

Authors: The Results section of the current paper was compared word-for-word with the Results section of our earlier publication:

You et al. Decreasing Birth Rate Determining Worldwide Incidence and Regional Variation of Female Breast Cancer.

The manuscript with track changes shows what have been amended. No full-length overlapping sentences exists.

In your revision ensure you cite all your sources (including your own works), and quote or rephrase any duplicated text outside the methods section. Further consideration is dependent on these concerns being addressed.

Authors: In reporting results of research using the same methods to address different topics using different data it is inevitable to repeat some phrases or words concerning statistical methods and results of their application. Thanks for your acknowledgement, but we have still modified somewhat repeated portions of the text.

**Reviewers' comments:**

Reviewer's Responses to Questions

Comments to the Author

1. Is the manuscript technically sound, and do the data support the conclusions?

The manuscript must describe a technically sound piece of scientific research with data that supports the conclusions. Experiments must have been conducted rigorously, with appropriate controls, replication, and sample sizes. The conclusions must be drawn appropriately based on the data presented.

Reviewer #1: Yes

Reviewer #2: Yes

Reviewer #3: Yes

________________________________________

2. Has the statistical analysis been performed appropriately and rigorously?

Reviewer #1: Yes

Reviewer #2: Yes

Reviewer #3: Yes

________________________________________

3. Have the authors made all data underlying the findings in their manuscript fully available?

The PLOS Data policy requires authors to make all data underlying the findings described in their manuscript fully available without restriction, with rare exception (please refer to the Data Availability Statement in the manuscript PDF file). The data should be provided as part of the manuscript or its supporting information, or deposited to a public repository. For example, in addition to summary statistics, the data points behind means, medians and variance measures should be available. If there are restrictions on publicly sharing data—e.g. participant privacy or use of data from a third party—those must be specified.

Authors: A whole set of data has been attached as an Excel file.

Reviewer #1: No

Reviewer #2: Yes

Reviewer #3: No

________________________________________

4. Is the manuscript presented in an intelligible fashion and written in standard English?

PLOS ONE does not copyedit accepted manuscripts, so the language in submitted articles must be clear, correct, and unambiguous. Any typographical or grammatical errors should be corrected at revision, so please note any specific errors here.

Reviewer #1: Yes

Reviewer #2: Yes

Reviewer #3: Yes

________________________________________

5. Review Comments to the Author

Please use the space provided to explain your answers to the questions above. You may also include additional comments for the author, including concerns about dual publication, research ethics, or publication ethics. (Please upload your review as an attachment if it exceeds 20,000 characters)

**Reviewer #1: This a fascinating and worthwhile article.**

Databases

Please report the assessment of the data compilers of the original documents and any technical reports on how the data on household size was collected and the reliability and validity of the measurement of household size.

Authors: This has been included in Section 2.2 of the manuscript. It reads:

*“In this document data sources are described as follows (Page 31): “The database comprises estimates of household size and composition obtained through analysis of microdata from the following data sources: Demographic and Health Surveys (dhsprogram.com), European Union Labour Force Surveys (http://ec.europa.eu/eurostat/web/microdata/european-union-labour-force-survey) and microdata samples maintained by Minnesota Population Center, Integrated Public Use Microdata Series, International: Version 6.4 [dataset]. Minneapolis, MN: University of Minnesota, 2015. http://doi.org/10.18128/D020.V6.4. Selected estimates of the average household size and headship rates were obtained through secondary sources, including the Demographic Yearbook of the United Nations (https://unstats.un.org/unsd/demographic/ products/dyb/dyb_Household/dyb_household.htm) and published reports of censuses. ”*

Similarly please comment in detail how the diagnosis of dementia death was made in the various countries you reported and any reliability and validity data. Was it based on samples and if so what was the sample size?

Authors: *DMR estimates are integrated by the team of WHO Global Health Estimates considering the latest available mortality and cause distributions reported by each country and most recent information from various WHO programs for causes of public health importance.* *This increases the comparability of DMR between countries. WHO website published the description of data, methods and cause categories in a Technical Paper [1].*

*This paragraph has been included in Section 2.2.1.*

What proportion of individuals and what are the numbers of individuals who were diagnosed with dementia in the countries you assessed? Some less developed countries have fewer people in the relevant age groups so the confidence intervals if based on samples would be wider.

Authors: See above.

Please comment in detail on Table 4 and differences between countries for which you have an explanation.

Authors: *The comments about the differences between different country groupings have added into the text related to the Table 4:*

*Small sample sizes in some categories of countries make differences between correlation coefficients found for them statistically insignificant and thus it is difficult to discern differences among those categories in the strength of correlations. However, in all categories, correlations are negative and often significantly different from zero. In economically developed country groupings, such as in the World Bank High income economics, WHO European Region and OECD all correlations are significant and moderately strong. Lower values of correlation coefficients in some groupings, especially those of lower income categories in the World Bank income classifications, may be a result of less variability in variables correlated when total ranges of their variation are artificially curtailed by categorisation. Averages of household size and dementia mortality in variously categorised countries show a tendency of larger household size to be associated with smaller dementia mortality.*

My comments are directed to strengthening your paper.

**Reviewer #2: Well done!**

Authors: Thank you. We are glad that you like this topic and this article.

The thematic is very important and we know that is a lack of evidence on this particular subject related to Large households/families to prevent dementia.

I have some considerations to the article that should be clarified

Abstract: Please mention the type of study design such as cross-sectional/correlation design.

Authors: This has been mentioned in the Methods in the Abstract: This is a retrospective cross-sectional study of correlations.

The method section should not have any of your results as in your last paragraph of method section.

Authors: The results have been removed from the section of Methods.

SES: This abbreviation did not mention before.

Authors: Socio-Economic Status has been explained and study design added to the Abstract.

Introduction:

1- It is easy to read, written in a professional way, and has plenty of information.

2- Many unnecessary conjunctions between paragraphs.

Authors: We read the Introduction again and again and have identified and removed the unnecessary conjunctions.

3- typos before reference (20, 21) in in small communities.

Authors: Corrected

4- Please can you add a paragraph regarding country grouping (low, middle, and high-income country). do you have statistics on the dementia rate?

Authors: The following paragraph includes how all the countries are grouped.

*The exploration into different correlations between household size and DMR also allowed us to compare the different levels of correlations in different country groupings. The criteria for grouping countries used the World Bank income classifications [2], WHO regions [3], countries sharing specific characteristics like geography, culture, development role or socio-economic status, like Asia Cooperation Dialogue (ACD) [4], Asia-Pacific Economic Cooperation (APEC) [5], the Arab World [5], Latin America and the Caribbean (LAC) [6], Southern African Development Community (SADC) [7] and Organization for Economic Co-operation and Development (OECD) [5]. All the population listings are sourced from their official websites for matching with the list of populations with DMR.*

*The country grouping mentioned specifically by the reviewer was described as “The criteria for grouping countries used the World Bank income classifications [2]----” and grouping criteria was referenced as well.*

We did include some epidemiology of dementia in the Introduction:

*“Worldwide, it is estimated that 5-8% of general population aged 60 and over have been diagnosed with dementia in past years [8].*

Methodology:

1- Study design?

Authors: The study design in this article is retrospective cross-sectional using all data reported by the international organizations. This has been include in Subsection 2.1 in the “Material and Methods”

2- The data source is not clear, need to clarify how it was done?

Authors: See answers to Reviewer 1 please.

3- The sample size is not well described - Power analyses?

Authors: The sample size equals number of countries. Sample sizes of our analyses are shown in all tables. This is stated in the text, too. For correlation coefficients (r, rho) the power of 0.80 is obtained with sample sizes of 194 for r=0.20, 176 for r=0.21, 160 for r=0.22 and 146 for r=0.23. Thus, for partial correlation coefficients between DMR and household size that are about 0.322, the power of 0.985 is obtained at the sample size of 159.

Results:

1- SMR variables???

Authors: SMR (standardized mortality ratio) was included as the dependent variable. The source has been described in Section 2.2.1.

2- Table 3. 0.000 replace it with 0.001, please add R change.

Authors: Thanks. 0.000 has been replaced with 0.001. Adjusted R^2^ have been inserted into Table 3 for visualizing the R change.

3- All abbreviations in all Tables should be mention as a footnote. Mainly Table 4

Authors: Done

**Discussion:**

1-The therapeutic effects of Oxytocin. Why you added. Is your study investigate the level of Oxytocin? You discussed previous studies regarding Oxytocin and its effect on dementia (VaD, FTD...) If not I suggest removing this or a better explain why you mention that in the first paragraph.

Authors: Discussion of the oxytocin was shortened and the title making it the separate subsection removed together with other subsection titles. Now oxytocin mentions are on a par with other interpretations in the Discussion.

2- You mention this statement: Similarly, five (5) studies conducted by Lambert and co-workers also identified the independent relationship between the meaning of life and family support among your people [116]. I returned to this paper and I read it carefully, Unfortunately, it is about the use of social media in healthcare settings not as you mention.

Authors: Thanks. Reference to Lambert and co-workers studies has been removed.

3- Can you add the year after you mention the authors such as Stutin and colleagues (??).

Authors: Thanks. This has been done.

4- Finally, the last paragraph regarding developed countries before the limitation section. I like the explanation, but to add more strength can you add more explanation between developing countries (Low- and middle-income) and developed countries.

Authors: At the end of the Discussion, we have added the following:

*“The fact that people live in bigger households in developing countries is a combined result of the economic situation and social customs. With lower incomes and less availability of housing, people are more likely to share accommodation while social customs demand respect and care for elderly people, who, in the situation of low incomes cannot afford to have living arrangements independent of members of younger generations. In developed countries higher incomes, greater availability of accommodation, higher mobility and individualistic lifestyles of adults, together with well-resourced retirement situations of older persons allow individuals or couples to have separate households. In this situation, social interactions with other individuals become sporadic personal choices rather than a daily necessity.”*

5- Good luck.

Authors: Thanks a lot for your time on assisting us in improving this study. Hopefully, it will be by the editor for publishing soon.

**Reviewer #3:**

1) Please consider reversing the order of x-axis values so that it shows a negative association between household size and dementia mortality rates as reported in text.

Authors: This has been done, and the updated figure has been included in the text.

2) Please provide a table for the descriptive statistics of the variables used for the study: basic measures of central tendency and variability for the whole countries and by different country categorizations.

Authors: Descriptive statistics (mean, median, standard deviation, minimum and maximum) of the variables used in the study were inserted into Table 1.

3) The correlation between household size and DMR reported on page 7 is -.6034, but the correlation for “worldwide” reported in Table 4 is -.524. Please clarify the difference between these two numbers.

Authors: These differences are not a result of errors. They are produced by different methods of calculating correlation coefficients: power regression, Spearman’s non-parametric correlation, and Pearson moment-product correlation of log-transformed values. Importantly, the values of correlation coefficients obtained by the power regression and non-parametric correlation do not differ significantly (<https://www.psychometrica.de/correlation.html>). Similarly, Pearson correlation coefficient does not differ significantly from the non-parametric (rho) one although it is smaller than the correlation calculated in the power regression. This is due to logarithmic transformation of data for product-moment correlation being only an approximation to exact homoscedasticity. Using approximative (weaker) methods that still produce significant relationships does not invalidate conclusions that such relationships exist since more precise methods can only show them to be stronger.

On Page 7, we reported:

“Figure 1 shows the relationship between household size and DMR. The relationship is logarithmic with moderately strong correlation negative correlation (r = -0.6034, p < 0.001). …” and

“Spearman’s rank correlation showed that household size was in significant negative correlation to DMR (r = -0.579, p < 0.001). This strength and direction of relationship were similar and observed in Pearson’s r household size and SMR variables (r= - 0.524, p<0.001).”

4) The main research question is to assess whether large household has the inhibitory role in lowering the risk for the residents “to develop dementia”. However, the dependent variable of the study is DMR, which is different from “development of dementia”. Please clarify why DMR, not some other measures more closely measuring the development of dementia. Perhaps, Alzheimer's disease morbidity can be explored together to draw a bigger picture but not sure about data availability.

Authors: Dementia mortality rate (DMR) data are more available and reliable. Morbidities reports may depend on the precision of diagnoses, but some sufferers of milder conditions may not be reported regardless of the levels of medical services in different countries. Cause of death reports, while still prone to some diagnostic errors, are more consistent. We have removed the phrase “development of dementia” because it has been included in the mortality (death). The reason is that nobody can die of dementia without previously developing it!

5) The top part of Table 2 and the bottom part of Table 2 provide the same numbers. And the numbers on the first line of page 8 do not exactly match the numbers in Table 2. FYI, ageing is not significant at the .05 level of significance since the p-value reported in Table 2 is .051, which is >.050. Please double-check.

Authors: Thank you for detecting these inconsistencies. The bottom half of the Table 2 has been removed and the numbers in the text have been corrected. Additionally, the mention of the correlation with Ageing has been removed as it is not the key in this study.

6) Table 4 reports valuable information about the variation in the association between household size and DMR. But it seems that the negative association between household size and DMR is only observed among high income countries (-.623, n=43), which is contradictory to the main finding of the study (Table 3). The results for countries grouped based on various factors somewhat agree with this result. Please discuss potential sources of the discrepancy.

Authors: We have given the following answer, included in the text of the paper, to the Reviewer 1’s similar question:

*“Small sample sizes in some categories of countries make differences between correlation coefficients found for them statistically insignificant and thus it is difficult to discern differences among those categories in the strength of correlations. However, in all categories, correlations are negative and mostly significantly different from zero. In economically developed country groupings, such as in the World Bank High income economics, WHO European Region and OECD all correlations are significant and moderately strong. Lower values of correlation coefficients in some groupings, especially those of lower income categories in the World Bank income classifications, may be a result of less variability in variables correlated.”*

7) Also wondering if the negative association between household size and DMR controlling for ageing, GDP, and urbanization, is still observed for those countries with high income. It would be very informative if the regression results with all variables (household size, ageing, GDP, and urbanization) can be reported for each country group, especially based on World Bank income classification, as well.

Authors: We have included results of partial correlations controlling for GDP, Ageing and urbanisation, and averages and standard deviations of Household size and Dementia Mortality into Table 4. Indeed, the partial correlation coefficient (r= - 0.460) is significant in the High Income group of World Bank classification.

The potential explanations for the insignificant correlations in low income, low middle and upper middle income country grouping have been included in the text.

8) It is unclear why using three different categorizations for countries. Other than World Bank one, it seems that the other categorizations provide more heterogenous grouping than homogenous.

Authors: We are just using all categorizations available to illustrate our point and indeed the World Bank one is more socioeconomically homogenous. Averages of the household size and dementia mortality in those groupings illustrate the idea that larger household size is, however variably, generally related to lower dementia mortality (Table 4).

________________________________________

6. PLOS authors have the option to publish the peer review history of their article (what does this mean?). If published, this will include your full peer review and any attached files.

Authors: Thanks for the offer. We would like to include the full peer review and all attached files for publishing together with the article.

If you choose “no”, your identity will remain anonymous but your review may still be made public.

Do you want your identity to be public for this peer review? For information about this choice, including consent withdrawal, please see our Privacy Policy.

Reviewer #1: Yes: Roger E. Thomas

Reviewer #2: No

Reviewer #3: No

[NOTE: If reviewer comments were submitted as an attachment file, they will be attached to this email and accessible via the submission site. Please log into your account, locate the manuscript record, and check for the action link "View Attachments". If this link does not appear, there are no attachment files.]

While revising your submission, please upload your figure files to the Preflight Analysis and Conversion Engine (PACE) digital diagnostic tool, https://pacev2.apexcovantage.com/. PACE helps ensure that figures meet PLOS requirements. To use PACE, you must first register as a user. Registration is free. Then, login and navigate to the UPLOAD tab, where you will find detailed instructions on how to use the tool. If you encounter any issues or have any questions when using PACE, please email PLOS at figures@plos.org. Please note that Supporting Information files do not need this step.

In compliance with data protection regulations, you may request that we remove your personal registration details at any time. (Remove my information/details). Please contact the publication office if you have any questions.

**References:**

1.	World Health Organization, *WHO methods and data sources for global causes of death 2000-2016. Global Health Estimates Technical Paper WHO/HIS/IER/GHE/2018.3.* . 2018 Geneva.

2.	The World Bank. *Country and Lending Groups | Data*. 2015; Available from: <http://data.worldbank.org/about/country-and-lending-groups>.

3.	WHO. *WHO regional offices*. [11.26.2015]; Available from: <http://www.who.int>.

4.	Asia Cooperation Dialogue. *Member Countries*. Available from: <http://www.acddialogue.com>.

5.	Asia-Pacific Economic Cooperation. *Member Economies-Asia-Pacific Economic Cooperation*. [11.26.2015]; Available from: <http://www.apec.org>.

6.	The United Nations Educational Scientific and Cultural Organization. *UNESCO Regions-Latin America and the Caribbean*. 2014 [11.26.2015]; Available from: <http://www.unesco.org>.

7.	South Africa Development Community. *Southern African Development Community: Member States*. [18.06.2015]; Available from: <http://www.sadc.int>.

8.	World Health Organization, *Dementia fact sheet* in [*https://www.who.int/news-room/fact-sheets/detail/dementia*](https://www.who.int/news-room/fact-sheets/detail/dementia). 2020, WHO: WHO Official Website.
