# Supplementary material for: Large household reduces dementia mortality: A cross-sectional data analysis of 183 populations
Source: PLoS One. 2022 Mar 3;17(3):e0263309. doi: 10.1371/journal.pone.0263309 (PMC8893634; doi:10.1371/journal.pone.0263309)
Supplement: Attachment — Submitted filename: 1 to 1 Response to Reviewer 1 comments.docx [file pone.0263309.s003.docx]

Dear Dr Wen-Wei Sung,

Thanks for the opportunity to address the reviewer’s further comments.

We have read the comments from the three reviewers, and found that only Reviewer 1 raised some issues, which are confusing and unnecessary. This is mostly likely due to his skip reading our manuscript and did not understand our manuscript in full.

Also, it seems that Reviewer 1 does not understand ecological study approach and data analysis very well.

Enclosed is our response to Reviewer 1’s comments.

If you have any further issues, please let us know.

Best regards,

Wenpeng YOU, PhDMED

Adjunct Senior Lecturer

Adelaide Medical School,

University of Adelaide

Adelaide, SA 5005

Australia

[Wenpeng.You@adelaide.edu.au](mailto:Wenpeng.You@adelaide.edu.au) OR [wpyou@hotmail.com](mailto:wpyou@hotmail.com)

Mobile: 0428 289 328

Response to Reviewer 1 second round comments

Reviewer #1: As this is a data base study the vaildity and reliability of the data are key The authors have not provided any new data on the reliability and validity of their data bases.

Authors: In the past decades, our research group have referred to over 100 publications in health management area which are only based on the population level (ecological) data. These data are published by the international organizations, such as United Nations and its agencies. With the secondary data, we also have published over 20 studies in various reputable journals, including Plos One. The statement below will be included in Section 2.2 in the manuscript text to explain the data reliability and validity:

The international organizations, the United Nations, WHO and the World Bank have monitored and published the data on the population specific economic status, health risk factors and nutrition and diet intake for decades. These data have been assisting government policy-makers and funders to track and investigate priorities of health research and development based on public health needs, while ensuring that funds and resources are used to meet the priorities. Their data have been more and more used in academic area for identifying the relationships between population level health and their risk factors. For example, recently they were used to examine the relationships between nutrients and obesity [1-4], diabetes [5-8], and the relationship between natural selection and obesity [9, 10]; type 1 diabetes [8] and cancers [10-12] respectively.

In the discussion, we have provided a short paragraph stating that our results present the correlations between published data, not necessarily causal relationships. The paragraph reads:

Due to the inherent uncertainties of the worldwide estimates provided by the WHO and the World Bank, our results must be seen as showing relations between published estimates, not necessarily precisely measured actual phenomena. The relations, however, are the closest available approximation of actual situation.

Page 2 They provide a reference to the WHO mortality rate, WHO Technical Paper [ref 43) but do not provide key data from that report.

Authors: The locations of the data on the number of deaths due to Alzheimer’s and other dementias and population size have been have be identified with the code numbers and line numbers in the manuscript as per the report:

The comprehensive and comparable assessment of country specific number of deaths due to “Alzheimer disease and other dementias (GHE code 950, Line 140)” and the country specific total population (ISO-3 Code) were provided by the WHO Global Health Estimates [13, 14].

Page 5 Please comment on data validity and reliability and missing data

Authors: The data validity and reliability has been addressed above. In terms of the missing data.

The United Nations and its agency have different approaches for defining the countries and territories, and not every county/territory supplies their data for every data collection round. This has made that, in the particular analyses, the number of populations (sample sizes) included may have differed somewhat because not all information was not uniformly available for all countries/territories.

We have commented that the data we used are the best available estimates. Please prove us wrong if you can. We have now stated that the data are missing for any country whose population was less than 90,000 in 2016.

Page 6 You refer to the World Bank published data on GDP and urbanisation but do not explain in detail the potential confounders you mention.

Authors: This is a data analysis-based epidemiology study about the risk factor for dementia. As the default in this stud approach, we acknowledged that socioeconomic level, life expectancy and urbanization are well established risk factors for dementia. In addition to these three factors, we are arguing that, potentially, decreasing household size is another risk factors which competently/confoundingly contributes to dementia incidence worldwide. The confounding effects of the three risk factors were removed through the partial data analysis in this study. This means that decreasing household size is a separate risk factor for dementia incidence.

Except for GDP and urbanisation mentioned the reviewer, ageing (measure by Life e(60)) has also been included as the confounder in our data analysis. This has been specifically mentioned in following two subheadings in Section 2.2 Data sources to specify why we have already included GDP, Urbanization and Life e_(60)_ in this study:

*3. The WHO published life expectancy at 60 (Life e(60)) was selected as a potential confounder [15].*

*4. The World Bank published data on Gross Domestic Product (GDP) and urbanization were also included as the potential confounder [16].*

we have explained reasons for considering GDP and urbanisation as confounders already in the text read by the reviewer. Now we have added another sentence. We think that our explanations are sufficient. This paper is not about details of data on well- known economic indicators (GDP and Urbanization). it is not a paper on economics and international relations which require the analysis of such data.

Page 6. "certain criteria for completeness and quality" Please provide details and how this affects your study
Authors: “certain criteria for completeness and quality” is the original quotation from the WHO Technical Paper expressing that the expected data quality and completeness have been met [17]. The set of data can be an effective reference for the governments when they are making their policies, funders when are considering the investment, and academics when they are performing their studies.

For this study, it is very positive to read this paragraph as it has given us the reassurance about the data quality and reliability.

Page 12 You wrote "Complied 2,200 years ago Huangdi Neijing" ... What are the data for the reliability and validity of the following statements?

"Large household promotes more mind-body interaction which offers biological protection" What is the evidence for this unsupported unsubstantiated statement.

Authors: The above statement has been re-written below with a proper reasoning, and this changed this definitive statement to a tentative paragraph:

Greater household/family has been explaining happiness in both Western and Chinese cultures [18, 19], and it has been quoted as an intervention for disease prevention and patient recovery. For thousands of years, happiness (Xi in Chinese pinyin) has been positively associated human health [20-23], and this mind-soul interaction is quoted as the key prevention for various diseases in “Huangdi Neijing” which was compiled 2,200 years ago and has influenced the medicine in south Asia.

Pleased read your manuscript carefully and remove any unsubstantiated statements, especially to be found in the Discussion section.

Authors: Thanks for this reminder. If there are any more unsustained found in the manuscript, please let us know.

**References:**

1.	Siervo, M., et al., *Sugar consumption and global prevalence of obesity and hypertension: an ecological analysis.* 2014. **17**(3): p. 587-596.

2.	Roccisano, D. and M. Henneberg, *Soy Consumption and Obesity.* Food and Nutrition Sciences, 2012. **03**(02): p. 260-266.

3.	You, W. and M. Henneberg, *Meat consumption providing a surplus energy in modern diet contributes to obesity prevalence: an ecological analysis.* BMC Nutrition, 2016. **2**(1).

4.	You, W. and M. Henneberg, *Meat in Modern Diet, Just as Bad as Sugar, Correlates with Worldwide Obesity: An Ecological Analysis.* J Nutr Food Sci, 2017. **6: 517**(4).

5.	Basu, S., et al., *The relationship of sugar to population-level diabetes prevalence: an econometric analysis of repeated cross-sectional data.* PLoS One, 2013. **8**(2): p. e57873: 1-8.

6.	Basu, S., et al., *Nutritional determinants of worldwide diabetes: an econometric study of food markets and diabetes prevalence in 173 countries.* Public health nutrition, 2013. **16**(1): p. 1-8.

7.	Weeratunga, P., et al., *Per capita sugar consumption and prevalence of diabetes mellitus – global and regional associations.* BMC Public Health, 2014. **14**: p. 186-91.

8.	You, W.-P. and M. Henneberg, *Type 1 diabetes prevalence increasing globally and regionally: the role of natural selection and life expectancy at birth.* BMJ Open Diabetes Research & Care, 2016. **4**(1): p. e000161.

9.	Budnik, A. and M. Henneberg, *Worldwide increase of obesity is related to the reduced opportunity for natural selection.* PloS one, 2017. **12**(1): p. e0170098.

10.	You, W. and M. Henneberg, *Cancer incidence increasing globally: The role of relaxed natural selection.* Evol Appl., 2017. **00:1–13**.

11.	You, W., et al., *Decreasing Birth Rate Determining Worldwide Incidence and Regional Variation of Female Breast Cancer.* Advances in Breast Cancer Research, 2018. **07**(01): p. 1-14.

12.	You, W., I. Symonds, and M. Henneberg, *Low fertility may be a significant determinant of ovarian cancer worldwide: an ecological analysis of cross-sectional data from 182 countries.* Journal of ovarian research, 2018. **11**(1): p. 68.

13.	World Health Organization, *Global Health Estimates 2016: Deaths by Cause, Age, Sex, by Country and by Region, 2000-2016*. 2018: Geneva.

14.	United Nations: Department of Economic and Social Affairs Population Division, ,, *World Population Prospects: The 2017 Revision, DVD Edition.* 2017.

15.	World Health Organization, *Healthy life expectancy (HALE)*. 2010.

16.	The World Bank. *World Bank Open Data*. 2016 12.07.2016]; Available from: <http://data.worldbank.org/>.

17.	World Health Organization, *WHO methods and data sources for global causes of death 2000-2016. Global Health Estimates Technical Paper WHO/HIS/IER/GHE/2018.3.* . 2018 Geneva.

18.	Angeles, L., *Children and Life Satisfaction.* Journal of Happiness Studies, 2009. **11**(4): p. 523-538.

19.	Nan, H., et al., *Psychometric evaluation of the Chinese version of the Subjective Happiness Scale: evidence from the Hong Kong FAMILY Cohort.* Int J Behav Med, 2014. **21**(4): p. 646-52.

20.	Bechara, A. and N. Naqvi, *Listening to your heart: interoceptive awareness as a gateway to feeling.* Nature neuroscience, 2004. **7**(2): p. 102-103.

21.	Cacioppo, J.T., et al., *Multilevel integrative analyses of human behavior: Social neuroscience and the complementing nature of social and biological approaches.* Psychological bulletin, 2000. **126**(6): p. 829.

22.	Damasio, A. and G.B. Carvalho, *The nature of feelings: evolutionary and neurobiological origins.* Nature reviews neuroscience, 2013. **14**(2): p. 143-152.

23.	Huangdi, Qibo, and Other ministers, *Huangdi Neijing*. Chapter 39 of Plain Questions: Disease prognosis, ed. Huangdi and Qibo. 2013, Wuhan: Wuhan Publishing House.
