# Supplementary material for: Large household reduces dementia mortality: A cross-sectional data analysis of 183 populations
Source: PLoS One. 2022 Mar 3;17(3):e0263309. doi: 10.1371/journal.pone.0263309 (PMC8893634; doi:10.1371/journal.pone.0263309)
Supplement: Attachment — Submitted filename: 1 to 1 response to review comments.docx [file pone.0263309.s004.docx]

**Comments to the Author**

1. If the authors have adequately addressed your comments raised in a previous round of review and you feel that this manuscript is now acceptable for publication, you may indicate that here to bypass the “Comments to the Author” section, enter your conflict of interest statement in the “Confidential to Editor” section, and submit your "Accept" recommendation.

Reviewer #1: (No Response)
Reviewer #4: All comments have been addressed
Reviewer #5: (No Response)
Reviewer #6: (No Response)
Reviewer #7: All comments have been addressed
 
2. Is the manuscript technically sound, and do the data support the conclusions?

The manuscript must describe a technically sound piece of scientific research with data that supports the conclusions. Experiments must have been conducted rigorously, with appropriate controls, replication, and sample sizes. The conclusions must be drawn appropriately based on the data presented.

Reviewer #1: No

Reviewer #4: Yes

Reviewer #5: Yes

Reviewer #6: Yes

Reviewer #7: (No Response)

3. Has the statistical analysis been performed appropriately and rigorously?

Reviewer #1: No

Reviewer #4: Yes

Reviewer #5: Yes

Reviewer #6: Yes

Reviewer #7: (No Response)

4. Have the authors made all data underlying the findings in their manuscript fully available?
The PLOS Data policy requires authors to make all data underlying the findings described in their manuscript fully available without restriction, with rare exception (please refer to the Data Availability Statement in the manuscript PDF file). The data should be provided as part of the manuscript or its supporting information, or deposited to a public repository. For example, in addition to summary statistics, the data points behind means, medians and variance measures should be available. If there are restrictions on publicly sharing data—e.g. participant privacy or use of data from a third party—those must be specified.

Reviewer #1: No
Reviewer #4: Yes
Reviewer #5: Yes
Reviewer #6: No
Reviewer #7: (No Response)

5. Is the manuscript presented in an intelligible fashion and written in standard English?

PLOS ONE does not copyedit accepted manuscripts, so the language in submitted articles must be clear, correct, and unambiguous. Any typographical or grammatical errors should be corrected at revision, so please note any specific errors here.

Reviewer #1: Yes
Reviewer #4: Yes
Reviewer #5: Yes
Reviewer #6: Yes
Reviewer #7: (No Response)

**6. Review Comments to the Author**

Please use the space provided to explain your answers to the questions above. You may also include additional comments for the author, including concerns about dual publication, research ethics, or publication ethics. (Please upload your review as an attachment if it exceeds 20,000 characters)
 
**Reviewer #1:**

Thank you for your updated version.

As I mentioned with your first submission the assessment of the risk of bias of the databases is crucial to your study. There are minimal changes in your manuscript. You have not assessed the risk of bias in your databases, which would have required identifying key documents about the methods they used and their assessments of reliability from the database constructors.

Authors: We have cited the “key documents about the methods” and “assessments of reliability from the database constructors”. Thus, we assessed, at least qualitatively, the risk of bias in databases we used. WHO databases, however still having some biases as it is impossible to avoid all errors, are the most reliable worldwide health databases. The best that can be used.

It is well known that each study methodology has their own inherent bias. For instance, data reporting inconsistency and inaccuracy is a major inherent bias for the studies which are based on the individual based data analysis. However, this is not an issue in ecological data as the data were collected in an objective way. For ecological studies, the critical bias is not the data reliability and validity, but how readership interpret the population/group level relationship at the individual level. In this study, we advanced that greater household size reduces dementia mortality at population level, but this might hold always true when readership applies this relationship at the individual level. This bias is also called “ecological fallacy”. This has been acknowledged and included in the section of limitation of the manuscript:

*“Secondly, the results based on the ecological approach in this work are subject to ecological fallacy.”*

*AND*

*“Due to the inherent uncertainties of the worldwide estimates provided by the WHO and the World Bank, our results must be seen as showing relations between published estimates, not necessarily precisely measured actual phenomena.”*

To highlight the nature of the ecological study, the title has been changed to:

*Large household reduces dementia mortality: A cross-sectional data analysis of 183 populations*

You have not considered all the possible confounders.

Authors: It is a common sense that none of data analysis based studies has considered or was able to consider “all possible confounders”. Most of individual data based studies only included one or two or even nil potential confounders due to data availability.

The advantages of ecological studies allow authors to include more potential confounders. With this advantage, we have considered three comprehensive potential confounders in our data analysis. These confounders may be the most likely, and the most common confounders which competed household size for contributing to dementia mortality.

You do not assess the implications of your data. For example you define aging as life expectancy at 60 and found the median is 19 years and the SD = 3.1 years. This implies a median age at death of 79, which would imply your population is heavily influenced by wealthier countries. The SD at 3.1 is small so can you draw conclusions based on this small SD?

Authors: Median life expectancy of 19 years at age 60 DOES NOT MEAN that the median age at death of the entire population is 79 years. This calculation by the Reviewer is a misunderstanding of the biometric parameters of the life table. The life expectancy at birth gives the “median age at death” of the entire populations with the full age range. The 19 years is the number of average years for the individuals who have already survived to age 60 years are going to live. They (60 years old) are expected to die at age 79 years, but this figure does not include ages of people who died before age 60, especially infants and children. Every demographer knows that average age at death of elderly people, so-called “normal age at death” is higher than the newborn life expectancy or the median age at death of any member of the population. Therefore, compared to SD for the Life e(0) or younger, the SD of 3.1 years within the age span above 60 years is not small at all (assuming normal distribution, which is not actually true). The SD =3.1 gives 18.6 years span of the entire age at death after 60 distribution, that is 60+19+/-9.3 range of median age at death is 69.7 – 88.3, while the CV is 16.3% (100*3.1/19). Certainly, conclusions can be drawn with this kind of variation since it will produce enough variance for regressions. Please note that the distribution of ages at death in the entire age range of humans is far from normal. It only approaches normality in the section of old ages, thus the definition of the “normal age at death”.

As you see from the above discussion, we have assessed implications of our data thoroughly. The senior author (Maciej Henneberg) is a paleodemographer who studied calculations and interpretations of biological parameters of life tables beginning in his PhD study and then throughout his career.

Similarly you found median household size was 3.9 with an SD = 1.4 Can you draw conclusions with such a small SD?

Authors: Here SD value is even greater than in the life expectancy discussed above: CV = 35.9% and the full range of the data distribution is 0-8.1. THE SD IS NOT “SMALL”. This statement of the Reviewer is sort of misleading.

I suggested that you remove undocumented and unsubstantiated statements but they remain.

Authors: We have read our text thoroughly again. Unfortunately, we could not find and identify “undocumented and unsubstantiated statements”. All our statements, even if not supported by a direct citation, are logical and based on general knowledge of the textbook kind that does not require citation. We would be more than happy to include the citations to supported statements which the reviewer considers unsubstantiated and undocumented.

You have found an important idea but have not proven your hypothesis with data of sufficient low risk of bias.

Authors: To advance novelties of this studies is the strength of the ecological study. I believe that there are no better data to advance and support such good study hypothesis. When you look at the dementia care service, almost all the care services for dementia patients can be linked to the beneficial effects of greater household size. As said, there is data to support a good is better than nothing done to support the good idea.

This sentence reads to us that Reviewer 1 is suggesting that our paper cannot be published because of the bias of data. However, his biased suggestion highlights his misunderstanding of our data selection and inadequate statistic knowledge. This may have led his/her to bias and over reaction to ecological data for ecological studies.

**Reviewer #4:**

The author responses well for the rebuttal and makes a great change for the revised manuscript. I have no further comment or suggestion for this manuscript.

**Reviewer #5:**

This ecological study showed that household size was an independent factor associated with dementia mortality at the population level, which provided evidence that social interaction may improve the prognosis of dementia.

The authors do their best to reduce any possible biases. I have no issue to highlight.

**Reviewer #6:**

The manuscript is well-written; however, I would like to clarify some of the points regarding the study:

**Major issues:**

1, In the introduction, the authors mentioned, “…., The above considerations directed us to try to identify possible contributing factors for dementia from the evolutionary perspective.” However, the current study seemed to focus on “whether smaller household could serve as a risk factor for people dying of dementia”, instead of “whether smaller household could serve as a risk factor for dementia”. Though the authors had avoided using the sentence “developing dementia” in the revised version, the quoted sentence might be confusing for readers, for the two aforementioned concepts are not exactly same.

Authors: Thank you. We have changed this.

I would recommend the authors provide a clearer explanation in the manuscript regarding the rationale of utilizing DMR manifesting the development of dementia.

Authors: Thank you. The rationale has been included in the section of 2.3 Data Selection.

*Greater household size not only shows its beneficial effects on protecting residents from developing dementia, but also offers its supporting or caring role in reducing the risk for patient to die from dementia. The dementia mortality rate could manifest the level of beneficial effect for dementia patient during their whole life span.*

2, Frailty bias could exist and should be addressed: Severe adverse statuses or comorbidities, such as cancer or organ transplantation could massively affect mortality, influencing risk for death occurrence. Could you please comment why the issue was not considered as one of the variables in the current study?

Authors: In this study, we advanced that greater household size may reduce dementia mortality rate. In order to show that small household size is another risk factor for dementia mortality. Statistically, we controlled the well-established existing risk factors (variables), such as socioeconomic level, lifestyle change and in particular ageing. Cancer and organ transplantation has not been well established well as the risk factor for dementia development and subsequent death after diagnosed with dementia. Like other data analysis based studies, we cannot include all the potential risk factors in the studies without considering their relevance and availability.

**Minor issues:**

1, For reference 43, according to the formal name of the document, I think the correct title of this technical paper should be “WHO methods and data sources for country-level causes of death 2000-2016”.(According to the WHO technical paper, retrieved from: <https://www.who.int/healthinfo/global_burden_disease/GlobalCOD_method_2000-2016.pdf>)

Authors: Thank you. We have changed this in the reference.

2, For information related to dementia mortality, the dataset utilized by the authors was based on Global Health Estimates 2016; however, currently the GHE report has been updated to 2019 version. Hence it might be difficult for the readers to directly find the dataset you have retrieved. Therefore, I would recommend the authors provide the exact website address to make readers more easily to access the key data identifying the number of dementia death and other information.

Authors: Thank you. This is a good idea. We have included the hyperlink on which we downloaded the data in the reference.

**Reviewer #7:**

(No Response)

7. PLOS authors have the option to publish the peer review history of their article (what does this mean?). If published, this will include your full peer review and any attached files.

If you choose “no”, your identity will remain anonymous but your review may still be made public.

Do you want your identity to be public for this peer review? For information about this choice, including consent withdrawal, please see our Privacy Policy.

Reviewer #1: No

Reviewer #4: No

Reviewer #5: No

Reviewer #6: No

Reviewer #7: No

[NOTE: If reviewer comments were submitted as an attachment file, they will be attached to this email and accessible via the submission site. Please log into your account, locate the manuscript record, and check for the action link "View Attachments". If this link does not appear, there are no attachment files.]
 
While revising your submission, please upload your figure files to the Preflight Analysis and Conversion Engine (PACE) digital diagnostic tool, [https://pacev2.apexcovantage.com/](https://pacev2.apexcovantage.com). PACE helps ensure that figures meet PLOS requirements. To use PACE, you must first register as a user. Registration is free. Then, login and navigate to the UPLOAD tab, where you will find detailed instructions on how to use the tool. If you encounter any issues or have any questions when using PACE, please email PLOS at [figures@plos.org](mailto:figures@plos.org). Please note that Supporting Information files do not need this step.

In compliance with data protection regulations, you may request that we remove your personal registration details at any time. (Remove my information/details). Please contact the publication office if you have any questions.
