# Supplementary material for: Large household reduces dementia mortality: A cross-sectional data analysis of 183 populations
Source: PLoS One. 2022 Mar 3;17(3):e0263309. doi: 10.1371/journal.pone.0263309 (PMC8893634; doi:10.1371/journal.pone.0263309)
Supplement: Attachment — Submitted filename: 1 to 1 response_ 02012022.docx [file pone.0263309.s005.docx]

PONE-D-21-26012R3

Large household reduces dementia mortality: A cross-sectional data analysis of 183 populations

PLOS ONE
__________________________________________________________________________________

6. Review Comments to the Author
Reviewer #1: The authors have made minimal changes. This is a retrospective databases study. Therefore, assertions of causal relationships cannot be made.

Authors: This had been listed as one of the study limitations (first).

1. The authors persist is making predictive statements:

Abstract: Thanks a lot for this comment.

The predictive statements have been toned down or deleted from the manuscript.

"Large households/families create more positive psychological well-being"

Authors: We stated that “Large households/families create more positive psychological well-being—-" in the Background of the Abstract. Now it is amended as:

*Large households/families may create more happiness and offer more comprehensive healthcare between the members. We analyse the protecting role against dementia mortality through examining the relationship between household/family size and dementia mortality rate at population level.*

This paragraph has been elaborated in the following paragraph copied from the manuscript:

*It is well researched that positive psychological well-being has been implicated in health across adulthood [1]. Household creates a social environment which is salient to maintain health for the co-residential members. On a daily basis, the individual members encounter this environment, play their social role and enjoy the social relations [2]. Moreover, studies also showed that large household offers the residents the subjective happiness [3] leading to low risk for residents to develop various cancers [4], for instance female breast cancer [5, 6] and ovarian cancer [7]. Subjective happiness was associated with mental health significantly stronger than with physical health in people with disabilities [8] and hospital patients [9]. A recent study revealed that greater household size has the protecting role against children developing mental health disorders [10].*

"Large household was an independent predictor."

Authors: This sentence was included in the Result of the Abstract below:

*Regardless of the contribution of ageing, socio-economic status and urban lifestyle to dementia mortality, large household was an independent predictor of dementia mortality (r = −0.331, p <0.001) in partial correlation analysis.*

Thanks for this comment. We found that we did not state this properly. We meant to say,

Small (NOT large) household was an independent predictor/risk factor for dementia mortality [independent of ageing, socio-economic status and urban lifestyle]. We apologize for this confusion.

Now it has been rewritten and included in the Abstract in consideration of the context:

*When we controlled for the contribution of ageing, socio-economic status and urban lifestyle in partial correlation analysis, large household was still in inverse and significant correlation to dementia mortality (r = −0.331, p <0.001). This suggested that, statistically, large household protect against dementia mortality regardless of the contributing effects of ageing, socio-economic status and urban lifestyle.*
Text

"large household protects against dementia mortality."

Authors: This sentence was in the Conclusion section of the Abstract:

*Independent of ageing, urbanization and GDP, large household protects against dementia mortality.*

Now it has been toned down:

*While acknowledging that ageing, urban lifestyle and greater GDP are associated with dementia mortality, this study suggested that, at population level, small household size was another risk factor for dementia mortality.*

large household "most significant predictor of DMR."

Authors: We stated that "most significant predictor of DMR." because of our data analysis results. In this study, standard multiple linear regression analysis was performed. This analysis model regressed multiple variables while simultaneously removing those that were not important/significant, but at the same time those most significant (important) predictors were maintained in order of their influencing effects. For instance, in Table 3 - Stepwise model, when household size was included as one of the predicting variables (four in total: household size, ageing, urban lifestyle and GDP), it became the most significant/important risk factor for dementia mortality rate.  We described this in the Result:

*However, when household size was included as an independent variable, household was selected as the variable having the greatest influence on DMR with R^2^ = 0.263, while ageing was placed second increasing R^2^ to 0.289.*

Before this, this analysis model was introduced in 2.4 Data analysis of the manuscript:

*3. Standard multiple linear regression (enter and stepwise) was performed to visualize the correlation between DMR and each predicting factor and identify the most significant predictor(s) of DMR respectively.*

2. The section on oxytocin.

In this section the authors assert that oxytocin is the mediator molecule between their assertions and refer to "a stream of studies..." Could the authors please provide detailed analyses of studies with serum levels and differences in serum levels of oxytocin with different household sizes and rates of interaction (which again would be correlational relationships and not causal) or delete this section on oxytocin. Neurobiologists would ask for better data than are presented here.

Authors: The paragraph discussing the mediating effects of oxytocin has been removed.

Much appreciated for providing this important comment from the perspective of neurobiologist. This makes this study reporting and discussion more rigorous. A stream of studies did introduce the therapeutic role of oxytocin on different diseases, but it seems that there is not enough substantiated scientific support, at least at the molecular level. The following two paragraphs have been deleted from the Discussion:

*Oxytocin is a hormone and a neurotransmitter that is associated with social bonding, such as empathy, trust, sexual activity, group bonding and relationship-building [11]. A stream of studies in the last decade reported that oxytocin release is not only associated with giving birth [12] and lactation [13], but also with daily interactions between non-kin household residents and/or family members, such as spouses [14-16], mother and children [17], father and children [18] and co-residential household members [19-21]. Oxytocin can keep family members and household residents happy and loyal to each other [22, 23], which may bring more positive psychological well-being to the family members. Regardless of cultural backgrounds [3], people from large household, especially from the same family have more life satisfaction [3, 24] which may lead to more oxytocin production within the hypothalamo-pituitary magnocellular systems. A self-reinforcing cycle is formed between more household interactions and more oxytocin production [11].*

*Oxytocin has been implicated in many aspects of social functioning. The therapeutic effects of oxytocin have not only been explored in dementia prevention and treatment, but also have targeted the treatment of diseases for other aberrant social behaviour related disorders [25], such as autism spectrum disorder [26, 27], posttraumatic stress disorder [28], schizophrenia [29], and anxiety disorders [30].*

3. Data elements.

The authors used DMR, "ageing" GDP and urbanisation as their variables. Could the authors please list potential known confounders (and potential confounders unknown in their database and control variables) which could affect their results.

Authors: In statistics-based epidemiology studies, those best-established contributing variables are included in data analysis models as the existing detrimental or beneficial factors for the studied health challenge. These factors may compete the studied (independent) variable for predicting the specific health challenge (dependent variable). As the health effects of these factors have not been examined in the clinical trials, the relationship between the health challenge between each of the best-established variables is potential or correlational (not causal). During the study result reporting and discussing, those best- established variables are generally called potential (due to their effects not confirmed in clinical trials) confounders (due to their competition with the specific studied independent variable).

In this study, our literature review showed that ageing, GDP and urban lifestyle are well established/known risk factors. However, the aim of this study is to advance small household/family size may be another risk factor for the increase of dementia mortality rate. In other words, small household/family size may exert some detrimental effects on dementia mortality except those contributed by ageing, GDP and urban lifestyle. We achieved this goal through data analyses in partial correlation when we statistically controlled for the three potential confounders (ageing, GDP and urban lifestyle), and in enter linear regression when we included all the four variables (household size, ageing, GDP and urban lifestyle).

Some factors could be the risk factors as well, such as hypertension, obesity, head injuries and hearing loss in middle life, and smoking, depression, diabetes and social isolation in later life. However, due to data availability and/or low levels of establishment as risk factors for dementia mortality in previous studies. We could not include them as the potential confounders in our data analyses. This question has led us to list an additional study limitation:

*Thirdly, dementia mortality has been associated with multiple risk factors which could confound the relationship between large household/family size and dementia mortality rate in this study. These factors include, but not limited to, hypertension, obesity, head injury and hearing loss in middle life, and smoking, depression, diabetes and social isolation in later life. However, these factors have not been well established for explaining dementia mortality. Also considering unavailability of the data on these factors at population level, we cannot include them as the potential confounders for ruling out their competition with the detrimental effect of small household/family size on dementia mortality in this study.*

Sorry, we did not collect and keep the data of the variables which were not considered as the major risk factors for dementia mortality rate.

Reviewer #6: The authors have fully addressed all raised issues, and I have no further major concerns.
However, it should be noticed that in the updated reference 41 (namely, the dataset of GHE utilized by the author), the website is revamped and it seems the original dataset is no longer available.
Hence, I think this should be stated in the manuscript to remind the readers that the version 2016 is no longer publicly accessible.

Authors: Thanks for bringing this important comment again.

With the assistance of Dr Cherian Varghese, the Coordinator, Management of Noncommunicable Diseases (MND), the hyperlink of the data source has been obtained:

[*https://www.who.int/healthinfo/global_burden_disease/GHE2016_Deaths_WBInc_2000_2016.xls*](https://www.who.int/healthinfo/global_burden_disease/GHE2016_Deaths_WBInc_2000_2016.xls)

It has been included in the References of the manuscript.

**References:**

1.	Howell RT, Kern ML, Lyubomirsky S: **Health benefits: Meta-analytically determining the impact of well-being on objective health outcomes**. *Health Psychology Review* 2007, **1**(1):83-136.

2.	Hughes ME, Waite LJ: **Health in household context: Living arrangements and health in late middle age**. *Journal of health and social behavior* 2002, **43**(1):1.

3.	Nan H, Ni MY, Lee PH, Tam WW, Lam TH, Leung GM, McDowell I: **Psychometric evaluation of the Chinese version of the Subjective Happiness Scale: evidence from the Hong Kong FAMILY Cohort**. *International journal of behavioral medicine* 2014, **21**(4):646-652.

4.	You W, Rühli FJ, Henneberg RJ, Henneberg M: **Greater family size is associated with less cancer risk: an ecological analysis of 178 countries**. *BMC cancer* 2018, **18**(1):924.

5.	Bai A, Li H, Huang Y, Liu X, Gao Y, Wang P, Dai H, Song F, Hao X, Chen K: **A survey of overall life satisfaction and its association with breast diseases in Chinese women**. *Cancer medicine* 2016, **5**(1):111-119.

6.	You W, Symonds I, Rühli FJ, Henneberg M: **Decreasing birth rate determining worldwide incidence and regional variation of female breast Cancer**. *Advances in Breast Cancer Research* 2018, **7**(01):1-14.

7.	You W, Symonds I, Henneberg M: **Low fertility may be a significant determinant of ovarian cancer worldwide: an ecological analysis of cross-sectional data from 182 countries**. *Journal of ovarian research* 2018, **11**(1):1-9.

8.	Van Campen C, Iedema J: **Are persons with physical disabilities who participate in society healthier and happier? Structural equation modelling of objective participation and subjective well-being**. *Quality of Life Research* 2007, **16**(4):635.

9.	Mukuria C, Brazier J: **Valuing the EQ-5D and the SF-6D health states using subjective well-being: a secondary analysis of patient data**. *Social Science & Medicine* 2013, **77**:97-105.

10.	Grinde B, Tambs K: **Effect of household size on mental problems in children: results from the Norwegian Mother and Child Cohort study**. *BMC psychology* 2016, **4**(1):31.

11.	Magon N, Kalra S: **The orgasmic history of oxytocin: Love, lust, and labor**. *Indian journal of endocrinology and metabolism* 2011, **15**(Suppl3):S156.

12.	Takayanagi Y, Yoshida M, Bielsky IF, Ross HE, Kawamata M, Onaka T, Yanagisawa T, Kimura T, Matzuk MM, Young LJ: **Pervasive social deficits, but normal parturition, in oxytocin receptor-deficient mice**. *Proceedings of the National Academy of Sciences of the United States of America* 2005, **102**(44):16096-16101.

13.	White‐Traut R, Watanabe K, Pournajafi‐Nazarloo H, Schwertz D, Bell A, Carter CS: **Detection of salivary oxytocin levels in lactating women**. *Developmental psychobiology* 2009, **51**(4):367-373.

14.	Carmichael MS, Humbert R, Dixen J, Palmisano G, Greenleaf W, Davidson JM: **Plasma oxytocin increases in the human sexual response**. *The Journal of Clinical Endocrinology & Metabolism* 1987, **64**(1):27-31.

15.	Carmichael MS, Warburton VL, Dixen J, Davidson JM: **Relationships among cardiovascular, muscular, and oxytocin responses during human sexual activity**. *Archives of sexual behavior* 1994, **23**(1):59-79.

16.	Gordon Jr G, Burch RL, Platek SM: **Does semen have antidepressant properties?** *Archives of Sexual Behavior* 2002, **31**(3):289-293.

17.	Kendrick KM: **The neurobiology of social bonds**. *Journal of neuroendocrinology* 2004, **16**(12):1007-1008.

18.	Weisman O, Zagoory-Sharon O, Feldman R: **Oxytocin administration to parent enhances infant physiological and behavioral readiness for social engagement**. *Biological psychiatry* 2012, **72**(12):982-989.

19.	MacDonald K, MacDonald TM: **The peptide that binds: a systematic review of oxytocin and its prosocial effects in humans**. *Harvard review of psychiatry* 2010, **18**(1):1-21.

20.	Van IJzendoorn MH, Bakermans-Kranenburg MJ: **A sniff of trust: meta-analysis of the effects of intranasal oxytocin administration on face recognition, trust to in-group, and trust to out-group**. *Psychoneuroendocrinology* 2012, **37**(3):438-443.

21.	Wudarczyk OA, Earp BD, Guastella A, Savulescu J: **Could intranasal oxytocin be used to enhance relationships? Research imperatives, clinical policy, and ethical considerations**. *Current opinion in psychiatry* 2013, **26**(5):474.

22.	Insel TR, Hulihan TJ: **A gender-specific mechanism for pair bonding: oxytocin and partner preference formation in monogamous voles**. *Behavioral neuroscience* 1995, **109**(4):782.

23.	Young LJ, Murphy Young AZ, Hammock EA: **Anatomy and neurochemistry of the pair bond**. *Journal of Comparative Neurology* 2005, **493**(1):51-57.

24.	Angeles L: **Children and life satisfaction**. *Journal of happiness Studies* 2010, **11**(4):523-538.

25.	Fineberg SK, Ross DA: **Oxytocin and the social brain**. *Biological psychiatry* 2017, **81**(3):e19.

26.	Parker KJ, Oztan O, Libove RA, Sumiyoshi RD, Jackson LP, Karhson DS, Summers JE, Hinman KE, Motonaga KS, Phillips JM: **Intranasal oxytocin treatment for social deficits and biomarkers of response in children with autism**. *Proceedings of the National Academy of Sciences* 2017, **114**(30):8119-8124.

27.	Alvares GA, Quintana DS, Whitehouse AJ: **Beyond the hype and hope: critical considerations for intranasal oxytocin research in autism spectrum disorder**. *Autism Research* 2017, **10**(1):25-41.

28.	Knobloch HS, Charlet A, Hoffmann LC, Eliava M, Khrulev S, Cetin AH, Osten P, Schwarz MK, Seeburg PH, Stoop R: **Evoked axonal oxytocin release in the central amygdala attenuates fear response**. *Neuron* 2012, **73**(3):553-566.

29.	Oya K, Matsuda Y, Matsunaga S, Kishi T, Iwata N: **Efficacy and safety of oxytocin augmentation therapy for schizophrenia: an updated systematic review and meta-analysis of randomized, placebo-controlled trials**. *European archives of psychiatry and clinical neuroscience* 2016, **266**(5):439-450.

30.	Dodhia S, Hosanagar A, Fitzgerald DA, Labuschagne I, Wood AG, Nathan PJ, Phan KL: **Modulation of resting-state amygdala-frontal functional connectivity by oxytocin in generalized social anxiety disorder**. *Neuropsychopharmacology* 2014, **39**(9):2061-2069.
